# The Simple Health System Rules That Create Value

**Published:** 2012-02-02

**Authors:** Thomas E. Kottke, Nicolaas P. Pronk, George J. Isham

**Affiliations:** HealthPartners Research Foundation; HealthPartners, Minneapolis, Minnesota; HealthPartners, Minneapolis, Minnesota

## Abstract

In 1991, Plsek sought to improve the quality of health care by challenging the readers of *Crossing the Quality Chasm* to find the few simple rules that might guide the local development of the 21st century health system. We have analyzed our health system's activities in the context of systems science as it seeks to create value (improve population health and patient experience, and reduce costs) for its stakeholders. We have concluded that 5 rules are simultaneously necessary and sufficient for success: 1) The stakeholders agree on a set of mutual, measurable goals for the health system; 2) the extent to which the goals are being achieved is reported to the public; 3) resources are available to achieve the goals; 4) stakeholder incentives, imperatives, and sanctions are aligned with the agreed-on health system goals; and 5) leaders among all stakeholders endorse and promote the agreed-on health system goals.

## Plsek's Challenge

The 2001 Institute of Medicine (IOM) report, *Crossing the Quality Chasm,* defined the ideal health system as one satisfying 6 attributes (it is safe, timely, effective, efficient, equitable, and patient-centered) and 10 operating rules (1):

1. Care is based on continuous healing relationships.
2. Care is customized according to patients' needs and values.
3. The patient is the source of control.
4. Knowledge is shared, and information flows freely.
5. Decision-making is evidence-based.
6. Safety is a system property.
7. Transparency is necessary.
8. Needs are anticipated.
9. Waste is continuously decreased.
10. Cooperation among clinicians is a priority.

In an appendix to the report*,* Plsek made a compelling argument that the solution to creating a health system that behaves as desired lies in perceiving it as a complex adaptive system governed by simple rules rather than as a complicated mechanical system controlled by myriad rules of governance (2). To operationalize the vision of the IOM report, Plsek challenged readers to identify these rules.

## Understanding the Behavior of Systems

Systems science offers hints about what the design rules for health systems that create value (ie, improve the health of the population, improve patient experience, and reduce costs [3]) might be. Senge observed that all systems have several components, each of which is necessary for the system to operate; together, they are sufficient for system operation (4). For example, despite the seemingly endless diversity among vehicles — big cars, small cars, trucks, tractors, ox carts, airplanes, or trains, to name a few — all have 4 components in common: 1) a propulsion component, 2) a source of energy, 3) a braking component, and 4) a steering component. Each one of these components is necessary and, together, the components are sufficient to operate the vehicle. If Plsek (2) and Senge (4) are correct, a health system that creates value can also be defined by a limited number of necessary components or rules that must be satisfied for the system to perform as desired. If all are satisfied, the system will create value. If even 1 component is missing, however, the system will not.

Common pool resources (CPRs) are a particular form of complex adaptive systems. CPRs are characterized by the difficulty of excluding people from using them and the fact that use by 1 person or group means that fewer resources are available for use by others (5). Examples of CPRs include groundwater resources, fishing grounds, and shared pasturelands. A health system meets the definition of a CPR.

Whereas the operating rules varied widely among the more than 5,000 CPRs identified by Ostrom (5), 7 design principles ("simple rules") characterized all of the robust CPR institutions that she studied:

1. Both the boundaries of the CPR and the people or organizations who have rights to withdraw from the CPR are clearly defined.
2. Appropriation rules (taking resources from the CPR) and provision rules (contributing to the maintenance of the CPR) are congruent with local conditions.
3. Most people affected by the operating rules of the CPR can participate in modifying the operating rules.
4. Monitors, who actively audit CPR conditions and behavior, are accountable to the appropriators (people who withdraw resources from the CPR) or are the appropriators.
5. Appropriators who violate operating rules are likely to be sanctioned.
6. Appropriators and their officials have rapid access to low-cost local arenas to resolve conflicts.
7. The rights of appropriators to devise their own institutions are not challenged by external governmental authorities.

An additional principle applies to large, complex CPRs: for CPRs that are part of larger systems, appropriation, provision, monitoring, enforcement, conflict resolution, and governance activities are organized in multiple layers of a nested enterprise (5).

These general design principles suggest the specific rules that must be satisfied for health systems to create value. The current health system in the United States fails to satisfy several of these rules.

Responding to the challenge Plsek put forth in the IOM report, we describe in this article the rules that we believe are necessary and sufficient to follow if health systems are to create value. Although we use examples from our integrated health system, HealthPartners, we believe that the rules apply to smaller units and to the US health system as a whole.

## HealthPartners' Search for Value

HealthPartners, located in the upper Midwest and headquartered in Minneapolis, Minnesota, is an integrated health system that finances care, administers benefits, provides care in hospitals and multispecialty clinic systems, and contracts for care with a large care network (www.healthpartners.com/public). The health system's mission and vision statements focus on optimizing health and creating value. In the past 10 years, HealthPartners has striven to create a system that satisfies the 6 attributes and 10 operating rules that *Crossing the Quality Chasm* used to define the ideal health system.

In 2008, HealthPartners adopted the Triple Aim, an idea proposed by Berwick et al (3), as its definition of value — the simultaneous improvement of the patient experience and population health and a reduction in the cost of care. For both disease prevention and disease treatment programs, HealthPartners uses the Triple Aim to guide organizational initiatives, focusing on those that are most likely to advance achievement of the 3 goals. Although it may seem daunting to simultaneously improve patient experience and population health while reducing costs, HealthPartners has done it for the health system as a whole (6) and for its employer-sponsored worksite health promotion programs (7).

Concurrent with efforts to implement the vision described in *Crossing the Quality Chasm* and increase the value of the services it delivers*,* HealthPartners has improved its quality of care. For example, clinics across the entire health system have been able to increase the proportion of diabetes patients meeting all targets of care from 9% in 2004 to 38% in 2009 (6). For 5 common chronic conditions, the organization has the lowest rate of adverse medical events (eg, rehospitalizations) in the Disease Management Purchasing Consortium database of nearly 200 health plans and employers (8). HealthPartners has also reduced the relative cost of care by 13 percentage points — from costs that were 4% greater than the market average in the 4th quarter of 2004 to costs that were 9% less than the market average in the second quarter of 2009 (6). Patient satisfaction was preserved during these changes. At no time since 2006 have fewer than 97% of its clinic patients reported that they would recommend their clinic to others (6).

HealthPartners has also developed health promotion programs that give the employer-purchaser a positive return on investment. The employer-sponsored worksite health promotion programs have been able to improve employee health, satisfaction, and productivity while reducing overall health care costs for both employers and employees (7).

## Five Simple Rules that Create Value in Health Care

After analyzing the activities of HealthPartners in the context of systems science, we have identified 5 simple rules that generate value for the stakeholders involved — patients, health care professionals, suppliers of pharmaceuticals and devices, health plans, and purchasers of health care.

**Rule 1:** *The stakeholders agree on a set of mutual, measurable goals for the system.*

If 2 people begin driving a car in Kansas City, and one wants to go to New York while the other wants to go to Los Angeles, it is likely that both drivers will become increasingly unhappy as the trip progresses, no matter which route they choose. A similar problem has arisen in health care: dissatisfaction is widespread because stakeholders' goals for the health system are divergent; furthermore, the goals are frequently at odds with the public good.

To promote a shared vision of goals, HealthPartners sponsored the formation of the Institute for Clinical Systems Improvement (ICSI), a "neutral ground" where competing organizations collaborate to create shared guidelines and care processes (9), and participated in the creation of Minnesota Health Scores (10). To further support the development of fair and equitable monitoring programs, HealthPartners leaders assist in developing and implementing regional and national measures through organizations such as the National Quality Forum, the National Committee for Quality Assurance (NCQA), IOM committees, the United States Preventive Services Task Force, the Task Force on Community Preventive Services, the Carter Center, and the Wisconsin Population Health Institute's Mobilizing Action Toward Community Health initiative (11).

**Rule 2:** *The extent to which the goals are being achieved is reported to the public.*

Ostrom observed that stakeholder access to monitoring data is critical to the success of CPRs (5). Without transparency, there is no accountability, and accountability is necessary to ensure value. HealthPartners has published objective measures of medical group performance since 1992 (12) and collaborates with Minnesota Health Scores (10) to report performance measures.

**Rule 3:** *Resources are available to achieve the goals.*

Demanding performance without providing the necessary resources will not create change. Money is just one of the resources required to implement systems that create value. Innovation in health systems requires the development of new products, procedures, devices, and systems that support the delivery of care. HealthPartners was an early adopter of the electronic health record, worksite health promotion, and e-visits. By collecting data on race, offering same-day mammography (women due for a mammogram are offered one during their office visit), and implementing a patient-recruiting system, HealthPartners has been able to reduce disparities in mammography screening among racial/ethnic minorities to a rate below the NCQA goal (13). In an economy characterized by scarcity, HealthPartners has been able to implement many of these initiatives by redistributing resources rather than allocating new resources.

**Rule 4:** *Stakeholder incentives, imperatives, and sanctions are aligned with the agreed-on health system goals.*

Misaligned stakeholder incentives create goals for the stakeholders that are not consistent with a health system that focuses on creating value. HealthPartners has several initiatives under way to align incentives of all stakeholders with the Triple Aim. To align the incentives of employees and dependents with those of the employer and the health plan, HealthPartners implemented a program in which health promotion and health care are offered as a single benefits package designed to maintain and improve employee/dependent health and address employer cost concerns. Informed by evidence (14), this intervention has high levels of participation and engagement in health assessments and follow-up programs, reports year-over-year improvements in the health of the participating populations, and improves productivity. It has also generated a positive return on investment (7,15).

To align the incentives of clinicians, HealthPartners reports performance of medical groups (12); in 1996, it introduced economic rewards for outstanding care (16). HealthPartners is also implementing payment models based on total cost of care. To avoid a "medical arms race" while ensuring access to high-quality care in a group of communities with rapid population growth, HealthPartners has collaborated with a competing hospital and a competing clinic system to systematically develop necessary services in the northwest quadrant of the Twin Cities metropolitan area (17).

**Rule 5:** *Leaders of all stakeholders endorse, promote, and honor the agreed-on health system goals.*

When goals are not periodically revisited, reviewed, and defended, missions drift, and the goals of individual stakeholders can begin to dominate the agreed-upon system goals. Health systems are no exception. To maintain an organizational focus on the Triple Aim, HealthPartners prominently features its mission and vision throughout its facilities, in its strategic plan, and in all communications. HealthPartners requires all employees to commit to 6 promises to patients, families, and members (18). As HealthPartners developed its organizational goals for 2014, it gave each employee an opportunity to provide input, and each physician-employee had an opportunity to offer his or her views on the employer-employee compact. In addition, to promote a statewide focus on creating value, HealthPartners sponsored the formation of ICSI.

HealthPartners executives lead and participate in community endeavors that promote social capital without regard to advancing the organization's market position. The chief executive officer leads a program to promote economic growth, job growth, and education reform to reduce regional health, education, and economic disparities (19). The chief executive officer is also a co-convener of the Health Advisory Group for Twin Cities Compass (20), a research program that tracks social conditions in the Twin Cities and other regions of Minnesota.

## Conclusion

The simple rules we have identified are not much different from those identified in Grand Junction, Colorado (21,22), or by Ballard et al (23) or Corrigan and McNeill (24). The similarities are not surprising — all of these studies address the same problem. They did not, however, identify the set of rules that are both necessary and sufficient to create value in health care, which we do here.

We have answered Plesk's challenge to identify the simple rules that govern the health system. Unless these rules are identified and addressed during the "plan" stage of a plan-do-study-act cycle, health systems may become lost in the forest of health care regulations and the opportunity to launch new initiatives. In a complex adaptive system, a single solution will fail unless all of the necessary rules for system functioning are satisfied.

The nation has progressed in increasing the value of health care. As early as 1993, teams that participated in the Institute for Healthcare Improvement Breakthrough Collaborative program had reduced patient waiting times by 50%, employee absenteeism by 25%, intensive care unit costs by 25%, and hospitalizations for congestive heart failure by 50% (25). However, the US health system is not ideal; a significant proportion of Americans cannot access the health system because they do not have health insurance, and people who are able to access the health system cannot uniformly expect to receive the quality of care envisioned in *Crossing the Quality Chasm*.

Others may not agree with the rules as we state them. Their objections might range from minor quibbles to outright rejection. They may also identify more than 5 rules. Regardless, we believe that the most efficient way to create a health system that delivers value is to identify simple rules and use them to guide the attempts to provide value to health system stakeholders. To paraphrase Plsek (2), the goal is not to agonize over the rules and the words used to describe them but rather to generate a "good enough" list to use as a tool to identify gaps in knowledge while striving to create value through repeated cycles of process improvement. This is the model we have adopted as we strive to deliver high-value health services.
